# Prognostic Role of Prostate-Specific Antigen (PSA) in male patients hospitalized for COVID-19

**DOI:** 10.12669/pjms.41.10.12564

**Published:** 2025-10

**Authors:** Yasin Ceylan, Ismail Demir, Sebnem Calik, Ozden Yildirim Akan, Emre Yoldas

**Affiliations:** 1 Yasin Ceylan, Department of Urology, University of Health Sciences, Izmir City Hospital, Izmir, Turkiye; 2 Ismail Demir, Department of Internal Medicine, Health Sciences University, Bozyaka Education and Research Hospital, Izmir, Turkiye; 3 Sebnem Calik, Department of Infectious Diseases, University of Health Science, Izmir City Hospital, Izmir, Turkiye; 4 Ozden Yildirim Akan, Department of Internal Medicine, Health Sciences University, Bozyaka Education and Research Hospital, Izmir, Turkiye; 5 Emre Yoldas, Department of Urology, University of Health Sciences, Izmir City Hospital, Izmir, Turkiye

**Keywords:** COVID-19, Prostate-Specific Antigen, Mortality, biomarker, androgens

## Abstract

**Objective:**

In this study, we aimed to examine the relationship between Prostate-Specific Antigen (PSA) level and mortality and morbidity of COVID-19 disease.

**Methodology:**

Demographic characteristics, PSA levels, comorbidities and clinical outcomes of male patients hospitalized at Bozyaka Training and Research Hospital between May 2020 to October 2021 due to COVID-19 and who had PSA measurements within the last one year were retrospectively analyzed. Exclusions included recent urologic intervention androgen deprivation therapy and five alpha reductase inhibitors. Mortality and morbidity rates were compared between patients with high PSA levels and those with normal PSA levels according to age. Age-specific PSA values followed the Oesterling guideline. Statistical analysis used T-test, Mann-Whitney U and chi-square tests, with significant set at p < 0.05

**Results:**

Of 664 patients, 42.2% had normal PSA levels, 57.8% had high PSA levels. Overall mortality was 32.0%. Mortality was higher in high PSA patients than normal PSA patients. (38.0% vs. 26.1%, p=0.0012). Intubation rates were higher in the high PSA group than normal PSA group (50.5% vs. 36.8%, p=0.0004). Hospital stays were longer and SOFA and APACHE II scores were higher in high PSA patients.

**Conclusions:**

PSA elevation is linked to increased mortality and morbidity in COVID-19 patients. PSA level may serve as a biomarker for risk assessment in COVID-19 patients.

## INTRODUCTION

The World Health Organization (WHO) classified coronavirus disease 2019 (COVID-19) as a pandemic in January 2020. Many factors affect disease progression, including comorbidities and demographic factors. The ACE2 receptor plays a pivotal role in the entry of SARS-CoV-2 into a host cell and androgen hormones may enhance the expression of ACE2, particularly among male patients. This study will examine the association between prostate-specific antigen (PSA) levels and clinical outcomes in male patients with COVID-19, with the hypothesis that higher PSA levels are correlated with greater disease severity and mortality. On January 11, 2020, the World Health Organization (WHO) declared COVID-19 a pandemic following the discovery of pneumonia and acute respiratory distress syndrome in Wuhan, China.^1^

Exposure to the renin-angiotensin-aldosterone (RAAS) axis and Type II Transmembrane Serine Protease (TMPRSS2) enzyme expression mechanism play important roles in the pathway of Covid-related comorbidities in the body.^2^ In the setting of SARS-CoV-2 infection, the virus engages the viral S-protein with the serine protease TMPRSS2 to facilitate entry into the cell itself via the ACE2 receptor.^3^ In humans, ACE2 receptors serve as the channels through which the virus enters alveolar epithelial cells and vascular endothelial cells. The currently accepted theory speculates that high ACE2 receptor and TMPRSS2 protein may lead to a greater risk of infection for this population. The nature in which the viral spike (S) protein binds to the ACE2 receptor and the mode in which TMPRSS2 enhances spike protein-ACE2 binding suggests that males have a greater susceptibility to COVID-19.^4^

Transmembrane serine protease (TMPRSS2), which is also known as epitheliasin and belongs to the type II transmembrane serine proteases (TTSPs), possesses a trypsin-like endopeptidase activity. Plays an oncogenic role in prostate cancer as the fusion gene with ERG, and has also been demonstrated to be essential for the cellular entry of severe acute respiratory syndrome coronaviruses (SARS-CoV). TMPRSS2-ERG fusion, found in roughly 100,000 cases every year, is the most common molecular alteration (40-50%) in prostate cancer patients. TMPRSS2 level is increased in both primary and metastatic prostate tumors and positively correlates with Gleason score.^5^ TMPRSS2 is an androgen-regulated serine protease, whereas PSA is a glycoprotein with a molecular weight of 33 kDa that also functions as a serine protease. Elevated TMPRSS2 levels have been observed in high-risk prostate cancer (Grade 3-4) compared to low-risk cases (Grade 1-2). Moreover, the TMPRSS2 gene has been implicated in the aggressiveness and tumor staging of prostate cancer.^6^ Various studies have indicated that elevated PSA levels and TMPRSS2 have a positive correlation.^7^,^8^

Other organs such as the kidneys, prostate and intestines may also have abundant quantities.^9^ Signaling pathways of androgen hormones, contribute to transcription of the ACE2 receptor and TMPRSS gene. Prostate-specific antigen (PSA) is a glycoprotein with a molecular weight of 33 kD. It is produced by epithelial cells lining the prostate ducts and acini where it functions as a serine protease. PSA levels can increase in non-malignant conditions such as benign prostatic hyperplasia as well as in prostate cancer. Elevated levels of androgen hormones in the body are also correlated to increased PSA serum levels and this occurs concurrently with the increased activity of the ACE-TMPRSS2 enzyme.^10^ Thus, there is a possibility for a connection between the ACE-TMPRSS2 enzyme and serum PSA levels in men, which further implies a conclusion between PSA serum levels, a marker related to prostate tissue, with severity of COVID-19 illness.^11^ The purpose of the study is to explore a relationship between age-related PSA serum levels and COVID-19 considering the association of COVID-19 complications with ACE-TMPRSS2 activity, which is related to PSA serum levels.

## METHODOLOGY

This is a single-center, retrospective study that includes male patients hospitalized due to COVID-19 between May 2020 to October 2021 who had PSA measurements available within the past year. Exclusion criteria included androgen deprivation therapy, five-alpha reductase inhibitors, and recent urological interventions. Patients were grouped according to age-specific PSA values. Parameters such as mortality, intubation, length of stay, and severity scores (SOFA, APACHE II) were analyzed. Statistical significance was set at p<0.05.

Between May 2020 to October 2021, 2865 patients were hospitalized at Bozyaka Training and Research Hospital due to COVID-19 infection. Of these, 55.3% were male (n=1584) and 41.9% of male patients (n=664) met our inclusion criteria. in this retrospective study, PSA measurements obtained within the past year from eligible patients were analyzed Patients had a respiratory rate > 24/min, an SpO2 level < 93% on room air and had imaging that reported bilateral diffuse lung involvement. Retrospective analysis included the age, demographic characteristics, comorbidities, biochemistry parameters, length of stay in the intensive care unit and mortality rates. Patients who had five alpha-reductase, had androgen deprivation therapy for prostate cancer and who had invasive procedures to the lower urinary tract in the prior six months were excluded.

Mortality and morbidity rates were compared between patients with high PSA levels and those with normal PSA levels according to age. Since PSA levels do increase with age, we grouped our patients based on age specific PSA values. Age specific PSA values were calculated with the cut-off values created by Oesterling and colleagues.^12^ SOFA (Sequential Organ Failure Assessment) was a clinical scoring system used to measure the severity of organ failure in intensive care unit patients and is used to predict mortality risk.^13^ The APACHE score (Acute Physiology and Chronic Health Evaluation) on the other hand was used as a scoring system used to assess the severity of illness, prognosis, and mortality risk of patients in the intensive care unit and is used to predict the 30-day mortality risk of patients. It was calculated using parameters such as body temperature, mean arterial pressure, heart rate, respiratory rate, arterial pH or HCO_3_, serum sodium, potassium, creatinine, hematocrit values, white blood cell count, Glasgow Coma Score, age, and chronic organ failure.^14^

### Statistical analyses:

it was conducted using SPSS version 25.0. Analysis of data included independent groups t-test for the normal distribution data and Mann-Whitney U test for non-normal distribution data. The variables’ conformity to normal distribution was tested with Kolmogorov-Smirnov/Shapiro Wilk test. Pearson and Fisher’s Exact Chi-Square tests were used to evaluate categorical variables. A p value <0.05 was accepted to indicate statistical significance.

### Ethical Approval:

Our study was planned on 21/05/2020-21.01.2022 with the approval of Bozyaka Education and Research Hospital Ethics Committee (ethics committee decision number: 2020-222).

## RESULTS

The data of 2865 patients who were hospitalized in our hospital due to COVID-19 infection were evaluated. Of the patients, 1584 (55.3%) were male and 1281 (44.7%) were female. Data of 664 male COVID-19-positive patients who were admitted to our hospital and whose PSA levels were measured in the preceding year were considered for this study. The mortality rate was 32.9% (n=219). Intubation rate was 44.7% (n=297). Among the patients, number of patients with low-normal age-specific PSA levels were n=280 (42.2%) and patients with high PSA were n=384 (57.8%). Patients under 50 years of age constituted 9.6% (64) of the cases, 24.5% (n=163) were aged 50-59 years, 46.8% (n=311) were aged 60-69 and 19% (n=126) were aged 70 years and older. Out of 664 patients, 219 (33%) died. The causes of mortality were acute respiratory distress syndrome (ARDS), Multiple Organ Failure (MODS), Septic Shock, Thromboembolic complications such as Pulmonary Embolism, DIC, Cardiac Complications (myocardial infarction, myocarditis), Ventilator associated pneumonia, Candidaemia, bacteremia, acute renal failure.

On intergroup comparison, between elevated PSA and normal PSA patients, mortality rate differences were statistically significant (p=0.00163). Mortality in low-normal PSA patients was 26.1% (n=73) compared to high PSA patients, with mortality of 38% (n=146). In regression analysis, elevated PSA was found to be a significant risk factor for death among these patients. (OR=1.74; 95% CI: 1.242-2.437).

In a statistically significant way, the intubation rate for those with elevated PSA level was greater compared to that for those with low-normal PSA level (p<0.05). Intubation was done at a rate of 36.8% (n=103) in patients with low-normal PSA, while 50.5% (n=194) in patients with high PSA. Elevated PSA was seen as a parameter in assessing the risk of intubation for Covid cases (OR=1.75; 95% CI: 1.277-2.396).

Patients who had high levels of PSA were classified based on their age and in accordance with the PSA cut-off values corresponding to each age category (Table-III). Accordingly, differences were assessed for the mortality and intubation factors according to PSA levels in patients below the age fifty and patients above fifty. For the 50-59-year-olds, there was a statistically significant difference for mortality and intubation (p=0.0003 and p=0.0001 respectively), (pairwise comparisons included). Other age groups, including those under 50 years, did not have any statistically significant difference for mortality or intubation.

**Table-I:** Demographic and clinical features of patients (N=664).

Variables	Subgroups	Frequency (n)	Percentage (%)
***PSA Levels (ng/mL)***			
	Low-Normal	280	42.2
	High	384	57.8
***Age Groups***			
	<50	64	14.9
	50-59	163	30.1
	60-69	311	42.4
	≥70	126	12.6
***Mortality***			
	Ex (Deceased)	219	33.0
	Live	445	67.0
***Intubation***			
	Yes	297	44.7
	No	367	55.3
***PSA/Mortality Rate***			
	Low-Normal	73	26.0 (of 280)
	High	146	38.0 (of 384)
***PSA/Intubation Rate***			
	Low-Normal	103	36.8 (of 280)
	High	194	50.5 (of 384)

**Table-II:** Outcomes by PSA Level.

Outcome	Normal PSA (n=280)	Elevated PSA (n=384)	Total (n=664)	p-value	OR (95% CI)
Mortality, n (%)	73 (26.1%)	146 (38.0%)	219 (33.0%)	0.0012	1.74 (1.24-2.44)
Intubation, n (%)	103 (36.8%)	194 (50.5%)	297 (44.7%)	0.0004	1.75 (1.28-2.40)

**Table-III:** Age-Specific Analysis of Mortality and Intubation Rates.

Age Group	PSA Cut-off (ng/mL)	Patients, n (%)	Mortality Rate n (%)	Intubation Rate n (%)	p-value (Mortality)	p-value (Intubation)
<50 years	2.5	64 (9.6%)	15 (23.4%)	23 (35.9%)	0.780	0.620
50-59 years	3.5	163 (24.5%)	84 (51.5%)	97 (59.5%)	0.0003	0.0001
60-69 years	4.5	311 (46.8%)	156 (50.2%)	189 (60.8%)	0.052	0.048
≥70 years	6.5	126 (19.0%)	68 (54.0%)	81 (64.3%)	0.120	0.090

Pearson’s Chi-Square test; significant differences highlighted (p<0.05).

Biochemical variables found to differ satisfyingly were found in the hemoglobin, leukocyte, thrombocyte, d-dimer, fibrinogen, glucose, urea, ALT, creatinine, PT, APTT, CK, BT and CRP parameters. No statistically significant differences existed in regards to their LDH, AST, INR and procalcitonin parameters. A significant difference was seen in the SOFA and APACHE II scores among the PSA level groups with increased values noted in high age-specific PSA patients. There was also strong statistical significance concerning length of stay between the groups with respect to patients with high age-specific PSA values. (p=0.0001) (Table-IV).

**Table-IV:** Comparison of biochemical parameters according to age-spesific PSA levels in COVID-19 patients (N=664).

Parameters	High PSA (n=384)	Low-Normal PSA (n=280)	p Value
Hemoglobin	11.5±2.4	12.0±1.4	0.002
Leukocyte*	8650±3900	7900±2300	<0.0001
Thrombocyte*	232000±163000	205000±120000	<0.0001
D-Dimer*	1300.5±658.2	800.2±582.2	<0.0001
Fibrinogen	902.2±288.2	601.3±188.6	<0.0001
LDH*	141.2±228.8	139.1±71.9	0.882
Glucose*	107.2±79.2	99.2±33.6	0.004
Urea*	42.4±33.7	35.2±26.1	0.003
Kreatinin*	2.6±1.1	1.9±0.8	<0.0001
ALT*	64.4±36.4	52.2±25.5	<0.0001
AST*	92.4±50.5	87.7±21.0	0.143
PT*	10.3±12.4	9.3±7.1	0.226
APTT	35.4±14.2	27.5±3.6	<0.0001
INR*	2.1±2.8	1.8±2.1	0.132
CK*	440.0±503.1	345.5±42.1	0.002
Bilirubin-Total*	2.3±2.7	1.7±1.3	0.001
CRP**	300.2±91.3	282.1±32.3	<0.0001
Ferritin*	363.4±359.7	323.4±283.4	0.003
SOFA Score	9.1±3.6	7.3±1.7	<0.0001
APACHE II Score	15.4±4.2	14.1±3.4	<0.0001
Time of Stay*	9.2±4.0	6.3±4.8	<0.0001

Independent t-test was used and p<0.05 was considered significant.

* Mann-Whitney U test was used.

## DISCUSSION

In this study, relationship between PSA-levels and clinical outcomes of male patients with COVID-19 infection was evaluated. Our study revealed a link between COVID-19 infection and increased hospitalization, duration of stay and death among male patients with high PSA values for their age, compared to those with normal or low PSA levels. The findings align with the hypothesis that androgen-driven mechanisms, including ACE2 receptor activity and TMPRSS2 enzyme expression, may exacerbate COVID-19 outcomes in men.^3^,^15^ We believe that ACE receptor expression is more prominent in patients with elevated PSA levels relative to their age, which may result in increased mortality in more severe clinical cases. Mortality associated with elevated PSA levels was 38.0% compared to 26.1% in the normal PSA group (p = 0012). Intubation rates, as demonstrated in Table-III were also significantly increased with elevated PSA (50.5% vs. 36.8%, p=0. 0004). For the high PSA group, mean hospital stay was 9.2 days (normal low: 6.3). Regression analysis also demonstrates PSA as an independent risk factor for both mortality and intubation.

PSA is an important biomarker that can be easily measured in the blood of men. Our findings indicating that PSA is a useful prognostic marker in COVID-19 are consistent with studies supporting the prognostic benefit of simple laboratory tests in COVID-19. Parmaksız et al. demonstrated a prognostic relationship between uric acid levels and COVID-19.^16^ In their study, Toori et al. showed that the neutrophil-lymphocyte ratio (NLR) could be a biomarker associated with disease severity and mortality in COVID-19.^17^ Considering these results, PSA should also be considered as a potentially useful prognostic marker in COVID-19.

Risk variables such as gender, genetic composition, age and concurrent diseases influence the considerable and complex clinical profile of COVID-19. In addition to immutable risk variables such as age and gender, elevated cardiovascular disease risk factors include hypertension, diabetes, chronic obstructive pulmonary disease, renal failure and obesity, all of which have a significant impact on the condition’s prognosis and mortality.^18^ This finding indicates that androgen sensitivity or prostate health during this specific decade may be particularly influential COVID-19 outcomes. An important finding that took our attention is that prognostic value of PSA is statistically significant between age of 50-59. This finding could be related with majority of the participants were in this age group. On the other hand, our finding is supported by Frumer et al.^19^ On the contrary, Ruan et al. investigated the prostatic secretions and urine in COVID-19 patients and found no significant difference compared to control grup.^20^,^21^

Multiple comorbidities that accompany to current situation of older patients could have reduced prognostic value of PSA. Non-significance in younger patients (<50 years) might be due to their lower baseline PSA values or other androgen dynamics. Ideally, advanced age must be used for risk stratification separately in geriatric and non-geriatric cohort. Due to high susceptibility for COVID-19, men with an elevated PSA should be monitored over time. PSA testing might be a less complex, adjunctive marker to help stratify risk in male patients with COVID-19 and focusing on the middle-aged and older males. Although Daskivich et al. revealed that age-related comorbidities may reduce prognostic value of PSA for prostate-related conditions,^22^ we could not find another study evaluating the prognostic value of PSA in COVID-19 patients. Additional research is needed to more definitively determine whether androgen-modulating drugs (e.g., five-alpha-reductase inhibitors) are of benefit in high-PSA/DRE-p groups.

The RAAS-ACE receptor pathway is important in the pathophysiology of many comorbid disorders. Furthermore, the creation of the virus’s protein, the ACE receptor and the TMPRSS2 enzyme appears to represent a common pathophysiological route in how COVID-19 infections work.^23^ Androgens upregulate ACE2 and TMPRSS2, facilitating viral entry and replication. Similar pathophysiological pathways, linked to male androgenic hormones, increase the risk of Covid-19 infection in heart disease and other illnesses associated with the RAAS-2 receptor system.^24^ The bulk of Covid-19 deaths worldwide have occurred among male patients. In Spain, male patients had nearly double the mortality rate as female patients.^25^,^26^ Since Covid-19 affects male patients more negatively and more frequently, only male Covid-19 cases were included in our study. Men face a hormonal disadvantage because female hormones, particularly estrogen and progesterone, protect against cardiovascular disorders, whereas androgenic hormones increase the prevalence of these diseases.^27^ Genes on the X chromosome that boost the immune system and male sex hormones may cause ACE2 receptors to be expressed more, which may help explain why men and women are more likely to get COVID-19 infections.^28^

Androgenic hormones contribute to elevated levels of cardiovascular factors in men. Larger prostates and increased ACE2 receptor expression in prostate tissue, which coincide with elevated PSA values, directly link these hormones to increased ACE receptor expression.^29^,^30^ Elevated ACE2 receptor levels caused by male sex hormone synthesis may explain the gender discrepancies found in COVID-19.^31^ Other mechanisms that are believed to explain the relationship between increased PSA levels are systemic inflammation,^32^ hypoxia,^33^ vascular dysfunction^34^ and coagulopathy with increased D-dimers and fibrinogen.^35^ Change in biochemical parameters such as Hb, leukocyte, D-dimers, fibrinogen, CRP shows that an increase in PSA is correlated to inflammation and coagulopathy.

The mean hospital stay was shorter in the normal PSA group (9.0 days vs. 30.5 days, p<0.0001). High levels indicate high androgen activity that could worsen the chances for viral infection followed by trapping inflammation. This is reinforced by higher SOFA and APACHE II scores, as well as adverse biochemical parameters (e.g., high D-dimer, CRP) of the high-PSA group that are attributable to severe COVID-19. Together, these findings confirm that PSA may serve as a potential biomarker of disease severity. Diaz et al. reported that ACE gene expression and A1166C polymorphism were effectively confirmed in both groups using RT-PCR with gel electrophoresis, but that the ACE receptor polymorphism exhibited more of a tendency to occur among BPH & PCA patients than in controls. Recently, a genetic factor (ACE1R polymorphism) has been identified as contributing to the risk of both BPH and PCA.^36^

According to our study, individuals with high PSA levels had significantly higher odds of dying, presenting protracted lengths and requiring invasive procedures such as intubation. This conclusion that PSA levels are an acceptable marker of ACE2-TMPRSS2 enzyme expression in COVID-19 is reasonable. Montopoli et al. found that prostate cancer patients who were on androgen suppression therapy had less diagnoses of COVID-19 compared to those who did not get this treatment. The authors have postulated that androgen suppression drugs lowered body levels of androgens, thereby significantly reducing expression level of ACE2-TMPRSS2 enzyme.^37^

They also found ACEI to be a receptor through which they can inhibit the growth of cancer cells and tumors driven by this family of receptors using specific inhibitors. It just confirms the similar idea previously that men with high expressing age-specific PSA and ACE2 receptor are at increased risk to present clinical requiring ICU or death. The present study provides evidence of a linear correlation between the density of ACE2 receptor and expression level of ACE-TMPRSS2 enzymes as well as PSA levels. This investigation has shown that age-specific PSA values higher than the normal range are risk factors for intensive care and mortality in COVID-19 male adults. Serum PSA levels were substantially elevated in patients with COVID-19 and associated with increased deaths.

Our results show that PSA is a potential prognostic biomarker for COVID-19 patients. Since PSA test is easily accesible and routinely used, this is considered an important advantage for clinical practice. Elevation in PSA is believed to be potential tool for risk assessment of middle-age patients’ group in terms of mortality and intubation. Besides accessibility of PSA test, our study also has some other qualifications that separates it from other researches. First of all, this research was carried out with a large sample size (n=664) which makes the results more reliable. Use of real clinical data obtained from a single center provided clinically-compatible outcomes.

We also evaluated the patients by age-related cut off values, so we avoided misclassification which could have led to incorrect conclusions. Extensive laboratory testing helped us research the relationship between PSA and systemic processes. Despite these advantages, out study also has some limitations. The generalizability of the study was limited due to retrospective and single-center design of the research. Another limitation relatively low number of patients in some groups.

## CONCLUSION

Higher PSA levels may be related with higher mortality in Covid19 patients. This association may be linked to higher androgen levels in these patients. Further larger study cohorts and prospective studies included androgen levels are needed to clarify the relation between the PSA and mortality.

### Authors’ Contributions:

**YC:** Literature search, Critical review of the manuscript. **ID SC:** Management of the project, Conception and design of the study. **OYA:** Data acquisition and statistical analysis. Critical Review. **EY:** Writing the manuscript. All authors have read and approved the final version of the manuscript.
